# Mitochondrial cAMP signaling

**DOI:** 10.1007/s00018-016-2282-2

**Published:** 2016-05-28

**Authors:** Fan Zhang, Liping Zhang, Yun Qi, Hong Xu

**Affiliations:** 1 National Heart, Lung, and Blood Institute, National Institutes of Health, Bethesda, MD 20892 USA; 2 National Institute of Dental and Craniofacial Research, National Institutes of Health, Bethesda, MD 20892 USA

**Keywords:** Protein import, Drp1, BH3, Fission, mtDNA, AKAP, TFAM, PKA

## Abstract

Cyclic adenosine 3, 5′-monophosphate (cAMP) is a ubiquitous second messenger regulating many biological processes, such as cell migration, differentiation, proliferation and apoptosis. cAMP signaling functions not only on the plasma membrane, but also in the nucleus and in organelles such as mitochondria. Mitochondrial cAMP signaling is an indispensable part of the cytoplasm-mitochondrion crosstalk that maintains mitochondrial homeostasis, regulates mitochondrial dynamics, and modulates cellular stress responses and other signaling pathways. Recently, the compartmentalization of mitochondrial cAMP signaling has attracted great attentions. This new input should be carefully taken into account when we interpret the findings of mitochondrial cAMP signaling. In this review, we summarize previous and recent progress in our understanding of mitochondrial cAMP signaling, including the components of the signaling cascade, and the function and regulation of this signaling pathway in different mitochondrial compartments.

## Introduction

Mitochondria, unique organelles enclosed by a two-layered membrane, produce the majority of cellular ATP in the eukaryotic cells through oxidative phosphorylation (OXPHOS). They also house the biosynthetic processes for many of the cell’s building blocks, including lipids, nucleotides, amino acids, and heme. Besides their well-recognized roles in energy and intermediate metabolism, mitochondria are now accepted as an important nexus for signaling cascades involved in cell growth, proliferation, differentiation and death [1].

Mitochondria originated from the endosymbiosis of ancestral bacteria inside primitive eukaryotic cells, approximately two billion years ago [1, 2]. Over their long evolutionary history, the engulfed bacteria ceased to function as free-living organisms, becoming instead semi-autonomous organelles. The majority of the mitochondrial ancestor’s genetic content was either lost or transferred to the host nuclear genome [3], and only a small fraction was retained in mitochondria. A typical animal cell contains hundreds to thousands copies of mitochondrial DNA (mtDNA), each encoding 13 essential subunits of the electron transport chain (ETC), 2 ribosomal RNAs and 22 tRNAs required for protein synthesis inside the organelle. The majority of the approximately 1500 mitochondrial proteins are encoded by the nuclear genome, synthesized in the cytoplasm and imported into mitochondria after translation [4, 5].

Mitochondria cannot be produced de novo. Instead, their proliferation requires mtDNA replication and the addition of lipids and proteins to the existing organelles [6, 7]. Mitochondria also undergo constant fusion and fission to adjust their shape and numbers in cells [8, 9]. Fusion and fission are also critical for the positioning, movement and even destruction of mitochondria. Mitochondrial biogenesis (the growth and division of pre-existing mitochondria) and dynamics (movement and morphological changes) are highly plastic in response to the cell’s energy demand, to developmental cues and to environmental stimuli. The success of such symbiosis requires carefully orchestrated communications between the eukaryocyte host and the prokaryocyte organelle. In the past a few years, it has become apparent that these communications are mediated in great part by cAMP signaling, a universal pathway conserved in all cellular organisms.

In this review, we briefly describe the compartmentalized landscape of mitochondrial cAMP signaling and discuss its regulation and diverse functions. We summarize the established roles of cAMP-PKA signaling the outer mitochondrial membrane (OMM) in protein import, mitochondrial fission and apoptosis. We also discuss the recent evidence for cAMP signaling in the mitochondrial matrix, as well as its effectors in the regulation of OXPHOS and mitochondrial biogenesis. In addition, we present unresolved questions about intra-mitochondrial cAMP signaling that need to be addressed in the future. Finally, we speculate on the therapeutic potential of managing mitochondrial cAMP signaling for diseases linked to mitochondrial deficiencies.

## Cellular cAMP signaling and compartmentalization (Fig. 1)

cAMP, one of the first identified and most versatile second messengers, mediates diverse cellular responses to extracellular signals. Over the past decades, various mechanisms triggering the production of cAMP have been identified, as well as its downstream effectors. Upon the activation of membrane receptors, downstream transducers, mainly G proteins, activate adenylyl cyclases on the plasma membrane (TmACs) to convert ATP to cAMP [10, 11]. Intracellular cAMP can also be produced by soluble adenylyl cyclases (sACs) in response to bicarbonate, calcium and the change of ATP level [12–15]. The major downstream effector of cAMP signaling is protein kinase A (PKA), a heterotetramer consisting of two catalytic subunits and two regulatory subunits. cAMP binds to the regulatory subunits, which releases and activates the catalytic subunits [16, 17]. Activated PKA phosphorylates and activates cAMP response element (CRE)-binding protein (CREB), a transcriptional co-factor that initiates an array of transcriptional cascades involved in immune response, cellular metabolism and mitochondrial biogenesis [18–20]. Besides its role in transcriptional regulation, PKA phosphorylates and modulates the activity of ion channels [21–23], cellular motor proteins [24, 25] and many enzymes involved in intermediate metabolism [26]. Many signaling and regulatory proteins, such as phospholipase C (PLC) [27], protein kinase C (PKC) [28], phosphoinositide 3-kinase (PI3K) [29, 30] and inositol trisphosphate (IP3) receptors [31], are also regulated by PKA-dependent phosphorylation. These phosphorylation events intertwine cAMP-PKA signaling with other cellular messengers and signaling cascades, and provide multiple feedback loops further modulating cAMP signaling [32, 33]. Intracellular cAMP level is regulated by the balanced act of ACs and cyclic nucleotide phosphodiesterases (PDEs), which terminate cAMP-PKA signaling by hydrolyzing cAMP to AMP [34, 35]. Besides the main effector PKA, cAMP can also directly activate the exchange protein Epac [36], the cyclic nucleotide-gated channels [37] and the Popeye domain-containing proteins [38, 39]. Within a single cAMP cascade, ACs, PKA, other downstream effectors and PDEs are often tethered together by scaffold proteins, the A-kinase anchoring proteins (AKAPs), at distinct subcellular locations [16, 40]. The compartmentalization of these signaling proteins [41–44] not only promotes the efficiency of cAMP signaling transduction, but also allows the same second messenger, cAMP, to mediate diverse physiological responses.

**Fig. 1:**
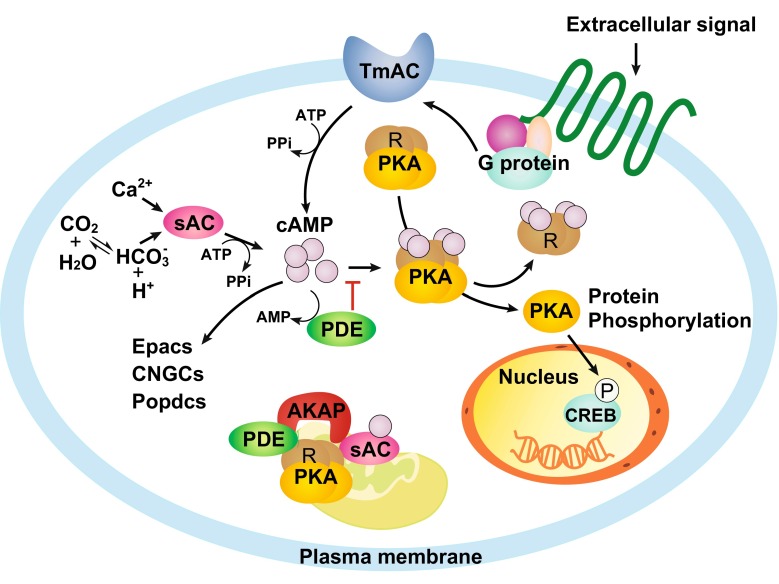
General cAMP signaling pathways. Intracellular cAMPs are generated by two classes of ACs, the transmembrane AC (TmAC) and the soluble AC (sAC). Both TmAC and sAC convert ATP to cAMP upon stimulus. TmAC can be activated by G proteins when an extracellular signal is received by the G protein-coupled membrane receptor. sAC is insensitive to G-proteins but can be activated by bicarbonate and calcium. Elevated cAMP level activates protein kinase A (PKA), the major effector of cAMP signaling, by releasing the two catalytic subunits (Cs) from the two regulatory subunits (Rs). Activated PKA in turn phosphorylates and activates numerous downstream protein targets, including the cAMP response element binding protein (CREB), a transcriptional co-factor regulating multiple cellular processes. Cyclic nucleotide phosphodiesterases (PDEs) are the negative regulators that terminate cAMP-PKA signaling by hydrolyzing cAMP to AMP. As a result, cAMP level and signaling activity are determined by the equilibrium between ACs and PDEs. In addition to its main effector PKA, cAMP can also directly activate the exchange protein Epac, the cyclic nucleotide-gated channels (CNGCs) and the Popeye domain-containing proteins (Popdcs). Within a single cAMP cascade, ACs, PKA, other downstream effectors and PDEs are often tethered together by an A-kinase anchoring proteins (AKAPs) at distinct subcellular locations

The compartmentalized structure of mitochondria [45] further shapes the cAMP signaling profile. The cAMP produced by TmAC or sAC in the cytosol can freely diffuse to the outer mitochondrial membrane (OMM) and activate the local PKAs at the mitochondrial surface. Moreover, as the OMM is readily permeable to ions and small molecules of 5 kDa or less [46], cytosolic cAMP might also regulate pathways inside the inter-membrane space (IMS). The inner mitochondrial membrane (IMM), however, is intrinsically impermeable due to a lack of Porins [47] and a high content of cardiolipin [48]. Nevertheless, cAMP is detected in the mitochondrial matrix of metazoans [49], which raises the questions of its origin in the matrix, and of its potential role in the regulation of biochemical pathways in this compartment.

## cAMP signaling in the outer-mitochondrial compartment (Fig. 2)

### Metabolic switch and mitochondrial protein import

As the interface between mitochondria and cytosol, the OMM is enriched in proteins that respond to intracellular signaling messengers regulating mitochondrial biogenesis, morphology and removal. Mitochondrial biogenesis entails the synthesis of approximately 1500 nucleus-encoded polypeptides in the cytoplasm and their import to mitochondria [4, 5]. The translocase of the outer membrane (TOM) complex represents the predominant pathway for importing proteins across the OMM [4, 5]. It is also one of the main downstream effectors of cAMP-PKA signaling tuning mitochondrial biogenesis to metabolism [50]. Three core components of the TOM complex, TOM70, TOM22 and TOM40, are phosphorylated by PKA in response to the glucose-induced cAMP increase triggered by a metabolic switch from respiratory to fermentable conditions [51–53]. Phosphorylation by PKA impairs the interaction between TOM70 and metabolite carrier/chaperone complexes (e.g., AAC/Hsp70) [51], inhibits the translocation of TOM22 to mitochondria [52] and prevents the integration of TOM40 into the OMM [53]. Thus, elevated cAMP-PKA signaling slows down the import of mitochondrial proteins, and fosters the metabolic switch from OXPHOS to glycolysis in conditions of increased glucose or reduced oxygen availability.

**Fig. 2:**
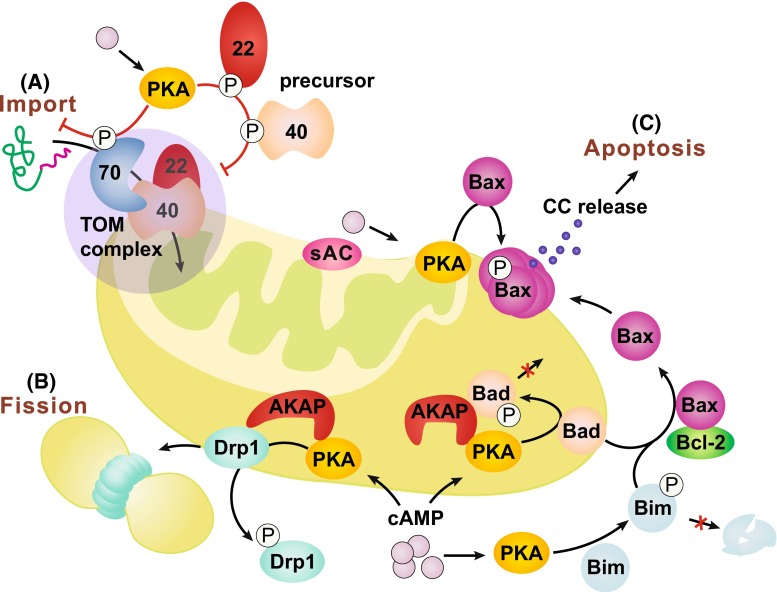
cAMP signaling in the outer-mitochondrial compartment. cAMP from the cytosol or produced by sAC on the OMM can activate the local PKA which in turn phosphorylates different targets associated with the OMM. **a** PKA phosphorylation impairs the receptor activity of TOM70 and its interaction with the metabolite carrier/chaperone, prevents TOM22 translocation and TOM40 integration into the OMM, and eventually slows down the import of mitochondrial proteins. **b** PKA phosphorylation can also block Drp1’s translocation to the OMM surface and thus lead to reduced mitochondrial fission. **c** In mammals, PKA phosphorylation inhibits Bad’s apoptotic activity but promotes Bim’s by increasing its stability against proteasome-dependent degradation. PKA phosphorylation of Bax promotes its translocation to mitochondria and triggers the release of cytochrome *c* (CC) and the maturation of the apoptosome, which eventually leads to apoptosis. AKAPs tether PKA and other proteins, e.g., Bad, on the OMM to facilitate the cAMP-PKA targeting. They also promote different signaling specificities under the same environmental context by providing a dynamic platform of proteins complex in multiple combinations

### Stress response and regulation of mitochondrial fission and fusion

Mitochondrial fission and fusion are indispensable for mitochondrial homeostasis [8, 9]. In a pool of overall healthy mitochondria, fusion mingles proteins, lipids and metabolites among individual mitochondria, thereby mitigating the defects of dysfunctional ones. On the other hand, fission enables the segregation of damaged mitochondria from a healthy population for their eventual removal, possibly through autophagy and mitochondrion-derived vesicles (MDV) [54–56]. Unbalanced fusion and fission can impair mitochondrial biogenesis, cause excessive mitochondrial fragmentation and trigger apoptosis [8, 9]. Elevated cAMP signaling upon bioenergetic stresses like starvation can inhibit mitochondrial fission, suspend unnecessary biogenesis and promote survival by sharing metabolites and boosting energy metabolism [57]. Mitochondrion fission is mediated by the dynamin-like GTPase, Drp1, which is recruited to the mitochondrial surface and assembled into a multimeric ring-like structure wrapping around the constriction points of the dividing mitochondria [58]. Phosphorylation of Drp1 by PKA blocks its translocation to the mitochondrial surface, leading to mitochondrial elongation rather than fission, which promotes cell survival [57, 59–61]. Reciprocally, Drp1 de-phosphorylation facilitates Drp1 recruitment to mitochondria and promotes fission, autophagy and apoptosis [54, 59, 62].

Under conditions of increased autophagy, a feedback response can promote fusion and survival by activating cAMP-PKA-dependent Drp1 phosphorylation and mitochondria elongation [57]. In addition to Drp1, PKA can also phosphorylate mitofusin 2 (Mfn2) [63] and bind to optic atrophy 1 (OPA1) [64, 65]. Both Mfn2 and OPA1 are involved in mitochondrial fusion [9]. However, the physiological significance of cAMP-PKA signaling in regulating mitochondrial fusion remains elusive.

### Apoptosis

Several apoptosis-related proteins are substrates of PKA [66–73]. In mammals, the intrinsic apoptotic pathway is regulated by the concerted action of anti-apoptotic Bcl-2 like proteins and pro-apoptotic BH3-only proteins [74, 75]. The pro-apoptotic proteins Bax [68, 69], Bad [66, 67, 70, 71] and Bim [72] can all be phosphorylated by PKA. However PKA phosphorylation on these proteins have completely opposite consequences: IL3-induced PKA phosphorylation of Bad inhibits its apoptotic activity [66], and the effect is mediated through the formation of PKA-Bad complex on the OMM [67, 70, 71]. By contrast, PKA phosphorylation of Bax promotes its translocation to mitochondria and triggers cytochrome *c* release and apoptosis [68, 69]. In addition, PKA phosphorylation of an isoform of Bim increases its stability against proteasome-dependent degradation and promotes its apoptotic effect [72].

### AKAPs as mediators of cAMP signaling specificity on the OMM

It is perplexing that a common messenger, cAMP, can elicit such diverse and distinct responses through different effectors on the OMM. It is now recognized that a family of PKA-anchoring proteins, the AKAPs, bind to and target PKA to distinct subcellular locations including the mitochondrial surface, the plasma membrane and the nucleus [16, 40]. AKAPs also act as scaffold proteins that tether PKA, PDEs, phosphatases, cytoskeleton proteins as well as other signaling molecules, such as PKC, MAPK, GSK-3β together, to form multi-protein signaling complexes [16, 40, 76–79]. Multiple AKAP isoforms have been found localized to mitochondria in metazoan [76, 78–86], and most AKAPs localize to the OMM. Specifically, D-AKAP1 has been proved directly siting in the OMM with its C-terminus protruding into the cytoplasm [87, 88]. The OMM-bound AKAPs tether PKA and other signaling molecules on the OMM [66, 78, 79, 84, 89–92]. This spatial organization allows the signaling specificities of different PKA pathways with a shared common messenger, cAMP, and thereby supports versatile combinations of mitochondrion related regulations. The sphingosine kinase interacting protein (SKIP) is an exceptional AKAP that recruits PKA to the IMS and the matrix of murine heart mitochondria [86].

## cAMP signaling inside the matrix (Fig. 3)

Mitochondrial biogenesis relies on the coordination between mitochondrial and nuclear genomes [6, 7]. In particular, the assembly of ETC complexes and mitochondrial ribosomes require the proper stoichiometry of nucleus- and mitochondria-encoded components. Mitochondria originated from bacteria, and bacteria use cAMP signaling to regulate motility, metabolism and DNA replication [93]. One can therefore speculate that a local cAMP signaling might have been retained in the mitochondrial matrix during evolution even though most mitochondrial components became encoded in the nuclear genome. Considering the emerging roles of cAMP signaling in the regulation of nuclear transcriptional activity and protein import into mitochondria, it is conceivable that cAMP signaling might act in parallel inside the matrix to modulate mitochondrial activities locally. Indeed, accumulating evidence supports a role for cAMP signaling inside the matrix as a necessary complement to the nuclear regulation.

**Fig. 3:**
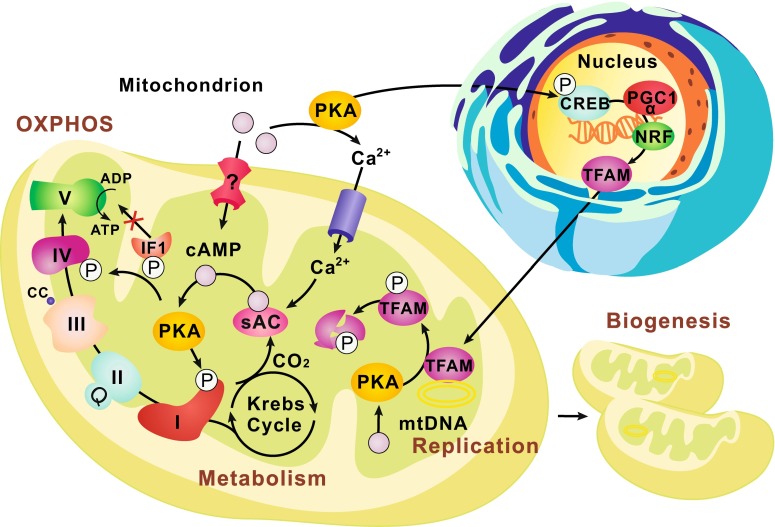
cAMP signaling in the mitochondrial matrix. The intra-mitochondrial cAMP-PKA pathway has been proposed to fine-tune metabolism by directly regulating the TCA cycle and respiration. sACs have been found in the mitochondrial matrix and produce cAMP locally in response to the CO_2_/HCO_3_^−^ generated by the TCA cycle. The matrix cAMP-PKA cascades are then activated, leading to the phosphorylation of ETC proteins such as Complex I and Complex IV subunits, modulating the OXPHOS and ATP production. In addition, PKA phosphorylation of the ATPase inhibitory factor 1 (IF1) abolishes its ability to bind to and inhibit Complex V. The sACs can also be activated by mitochondrial uptake of Ca^2+^. Active mechanisms for transporting cAMP into the matrix remain to be identified. To coordinate the OXPHOS and energy needs, the cAMP-PKA pathways outside mitochondria can activate the nuclear CREBs and the downstream transcription factors (PGC-1α, NRF) to promote TFAM (mtTFA) production, mtDNA replication and eventually mitochondrial biogenesis. On the other hand, the matrix cAMP signaling could exert a negative regulation on this process by increasing the PKA phosphorylation-dependent degradation of TFAM

### Origin of cAMP in the mitochondrial matrix

Although cAMP per se cannot freely diffuse across the IMM from the cytosol, both biochemical assays with isolated mitochondria [94, 95] and live-cell experiments using genetic reporters [49, 96, 97] have demonstrated the presence of cAMP inside mammalian mitochondria. However, the origin of matrix cAMP is still debated. After a brief incubation with purified mitochondria in vitro, cAMP readily accumulates in the mitochondrial matrix, suggesting that it could be actively transported into the matrix [94]. But recent studies using genetic cAMP reporters in cultured human cell lines (Hela, HEK293T) and primary rat cardiomyocytes argue that mitochondrial inner membrane is impermeable to cAMP [96, 97]. A model for generating cAMP locally has hence been proposed. It involves a mitochondrially localized sAC that can be directly activated by HCO_3_^−^ [12] and was shown to synthesize cAMP locally in response to the CO_2_/HCO_3_^−^ produced in the TCA cycle in mammalian cultured cells [95–100]. The matrix CO_2_-sAC-cAMP-PKA cascade is also involved in the allosteric regulation of COX activity by ATP in the *S. cerevisiae,* demonstrating a conserved role of cAMP signaling in fine-tuning energy metabolism [101]. However, the *Drosophila* and *C. elegans* genomes do not encode any known sAC [102], and the prevalence of intra-mitochondrial cAMP signaling in metazoans has been called into question.

We recently constructed a matrix-localized cAMP sensor by fusing the new generation of cAMP reporter, ICUE3, with SOD2, a bona fide mitochondrial matrix protein [103]. Using this construct, we demonstrated the existence of cAMP in the mitochondrial matrix of *Drosophila* cultured cells. Given the lack of sAC in the fly genome, our finding suggests that an unidentified mechanism allows the rapid entry of cytosolic cAMP into the mitochondrial matrix, which is consistent with a previous biochemical study [94]. Still, the *Drosophila* genome contains 14 genes that encode 38 different AC isoforms (http://flybase.org/). We thus cannot rule out the possibility that a matrix AC might be among them and produce cAMP locally. Although the sources of matrix cAMP may differ in different organisms and cell types, the presence of cAMP in the mitochondrial matrix now appears universal.

### PKA as the main effector of cAMP signaling in the mitochondrial matrix

Protein kinase A has long been considered as the main effector of intra-mitochondrial cAMP to regulate mitochondrial energy metabolism [95, 96, 104–107]. Large-scale phosphoproteome analyses revealed that many enzymes in the TCA cycle and ETC complex subunits were phosphorylated at the PKA consensus sites [108, 109]. These observations suggest that PKA is one of the most active kinases in the matrix [108, 109]. In support of this idea, 85 % of PKA activity associated with purified mitochondria is derived from the mitochondrial matrix fraction [110]. However, FRET-based PKA activity reporters that are targeted to the mitochondrial matrix do not respond to the addition of membrane-permeable cAMP analogs or bicarbonate [97]. It has been argued that the phosphorylation of matrix proteins at PKA consensus sites might take place before their import to the matrix [97]. We have developed a bimolecular fluorescence complementation (BiFC) assay for assessing the submitochondrial localization of proteins [103]. The assay is based on the fact that two halves of a GFP molecule will reconstitute into a whole, fluorescent GFP molecule only when they are in physical proximity [111]. By applying this sensitive assay, we demonstrated that PKA indeed locates to the mitochondrial matrix [103], substantiating the presence of local cAMP-PKA signaling.

### Other members of the cAMP signaling pathway in the matrix

Besides the sACs and PKA, other components of cAMP signaling cascade have also been identified in the matrix, including cAMP PDE activity proteins such as PDE2A2 [100] and Prune [103]. Interestingly, PDEs can be inhibited by hydrogen sulfide (H_2_S), which is produced in the matrix by enzymes such as 3-mercaptopyruvate sulfurtransferase (3-MST), cystathionine β-synthase (CBS) and cystathionine g-lyase (CSE) in response to environmental stresses like hypoxia [112]. These findings suggest a potential link between the environmental stresses and cAMP signaling in the mitochondrial matrix. In summary, almost a full complement of key components of the cAMP signaling cascade (CO_2_/HCO_3_^−^, Ca^2+^, sAC, cAMP, PKA, PDE and H_2_S) has been identified in the matrix, strongly supporting the existence of a distinct cAMP signaling in the mitochondrial matrix, which may have specific physiological and pathological implications.

### Intra-mitochondrial cAMP signaling regulates OXPHOS

A rich body of literature has documented the phosphorylation of ETC complexes and enzymes in the TCA cycle by PKA [95, 104–109, 113–116]. This observation suggests that the cAMP-PKA pathway in the matrix may fine-tune metabolism by directly regulating the TCA cycle and respiration. A rise in CO_2_ or bicarbonate concentration is thought to activate a matrix-localized sAC. The sAC produces cAMP locally, which in turn activates PKA and leads to phosphorylation of cytochrome *c* oxidase subunit IV isoform 1 (COXIV-1), resulting in enhanced cytochrome *c* oxidase (COX) activity and OXPHOS [95]. In yeast, a similar phosphorylation-dependent regulation on Cox5a, the homolog of COXIV-1, has also been reported [101]. Evidences also suggest that PKA can phosphorylate subunits of Complex I, which increases Complex I activity and stability, and hence enhances respiration [107, 113, 117–122]. Given that CO_2_ is the end product of the TCA cycle, the cAMP-PKA pathway provides a feed-forward regulation on two modules of energy metabolism: TCA cycle and ETC complexes. Recently, Complex V activity has also been found under the regulation of matrix cAMP-PKA pathway [123–125]. Complex V (mitochondrial ATP synthase) catalyzes the synthesis of ATP through the proton gradient generated by the ETC complexes and executes the reverse hydrolysis when the membrane potential falls below a threshold (pH of ~6.7 or below) [126]. An ATPase inhibitory factor 1 (IF1) is thought to act as a “reverse rotation brake” for the ATP synthase motor, preventing the “wasteful” ATP hydrolysis under the anaerobic condition [126, 127]. A recent study demonstrated that IF1 binds to Complex V and inhibits not only its hydrolase but also its ATP synthase activity [123]. IF1 can be phosphorylated by PKA, which prevents it from binding to Complex V, and thereby relieves its inhibitory effects on both the hydrolytic and synthetic activities of Complex V [123]. Thus the phosphorylation status of IF1 appears to be key in regulating the flux of glycolysis and OXPHOS corresponding to certain physiological context [124]. Furthermore, De Rasmo and colleagues recently have demonstrated the importance of the sAC-cAMP signaling for the organization and activity of Complex V in isolated rat mitochondria and myoblast cultures [125].

The impact of PKA on ETC complexes appears to be multifaceted depending on the environment cues. Under hypoxia/ischemia condition, excess of reactive oxygen species (ROS) induces the sequestration of PKA catalytic α subunit into the matrix, and leads to the hyper-phosphorylation of Complex IV and reduced COX activity [114, 128]. PKA phosphorylation on several Complex IV subunits during heart failure may also inhibit OXPHOS by either disrupting the complex assembly or reducing its stability [129]. Taken together, the intra-mitochondrial cAMP-PKA signaling may serve as an acute and local mechanism allowing mitochondria to rapidly adjust energy output in response to environmental stress, and to oxygen and nutrient availability. Under the aerobic condition, the steady stream of CO_2_ produced from TCA cycle activates sAC-cAMP-PKA cascade, which in turn tunes up the activities of the ETC complexes by coordinating TCA cycle and ETC complexes. While CO_2_ production is diminished under metabolic or environmental stress, ROS can directly activate PKA to inhibit ETC complexes to avoid excessive ROS production. Of note, Ca^2+^ can also activate sAC, the principal source of matrix cAMP [96], highlighting a potential crosstalk and synergy between these two common second messengers (cAMP and Ca^2+^) in regulating energy metabolism.

### Intra-mitochondrial cAMP signaling regulates mitochondrial biogenesis

Regulating OXPHOS is not the only way through which mitochondria respond to the cellular and environmental cues. A long-term adaptation of energy homeostasis can be achieved by modulating mitochondrial biogenesis. As previously described, during stresses such as hypoxia and starvation, the cAMP-PKA pathway can suppress mitochondrial biogenesis by inhibiting protein import and mitochondrial fission. On the other hand, the cytosolic cAMP-PKA pathway can also activate the nuclear CREBs and the downstream transcription factors (PGC-1α, NRF), which in turn activates the transcription of mitochondrial transcription factor A (mtTFA, also abbreviated as TFAM) and mitochondrial biogenesis [6, 130]. CREBs are also found inside mitochondria, binding to the CREs on the mtDNA D-loop, and directly regulating mtDNA-encoded gene expression [131–134]. The translocation of CREBs into mitochondria may be facilitated by chaperones like mtHsp70 [132] or by a process that depends on both membrane potential and TOM complex [134]. Both nuclear and mitochondrial CREB pathways promote neuronal survival in the brain [132, 133, 135], which is consistent with their positive roles in mitochondrial biogenesis.

Matrix cAMP signaling could also exert negative regulations on mitochondrial biogenesis. Human mtTFA can be phosphorylated by PKA, leading to decreased mtTFA-mtDNA interaction and increased mtTFA degradation [136]. Recently, we confirmed such phosphorylation in *Drosophila* and found that Prune, a mitochondrial PDE, stabilizes mtTFA by down-regulating cAMP levels in the mitochondrial matrix, thereby promotes mtDNA replication [103]. It thus appears the cAMP-PKA signaling acts as a “double agent” to fine-tune the mtTFA level. It promotes the expression of nuclear-encoded mtTFA, while counterbalances this action by negatively modulating mtTFA protein level through PKA-dependent degradation in the mitochondrial matrix. Our finding demonstrates the prevalence of mitochondrial matrix cAMP signaling and provides a new insight into its role in coordinating nuclear and mitochondrial regulation on mitochondrial biogenesis.

## Future perspectives

### Remaining mysteries in the mitochondrial cAMP signaling pathways

The repertoire of cAMP-PKA signaling components identified both on the OMM and in the mitochondrial matrix is growing. New regulations on mitochondrial functions by cAMP-PKA signaling keep emerging. We now recognize that cAMP signaling can respond to extracellular stimuli, intracellular cues or even environmental stresses to modulate a variety of mitochondrial behaviors including energy homeostasis, mitochondrial biogenesis and cell death. The literature also demonstrates the great complexity of mitochondrial cAMP signaling. The compartmentalization of mitochondrial cAMP signaling ensures its specificity. PKA phosphorylation on the same protein under different environmental or physiological context can lead to distinct, even opposite functional consequences, further demonstrating the complexity of mitochondrial cAMP signaling. Meanwhile, cAMP signaling pathway in different mitochondrial compartments (the OMM and the matrix) can work synergistically, and may interact with cytosolic and nuclear cAMP signaling to achieve coordinated and balanced regulations on mitochondrial functions.

The discovery of intra-mitochondrial cAMP signaling in *Drosophila* not only demonstrates the prevalence of mitochondrial cAMP signaling in metazoan, but also reveals an unknown regulation on mitochondrial biogenesis. However the lack of sACs in *Drosophila* and *C. elegans* genomes [102] presents an unsettled issue regarding the source of mitochondrial cAMP in these organisms. It is worth exploring whether there are unidentified AC isoforms that localize to mitochondria in these species. Meanwhile it remains to be determined whether there is indeed an active transport mechanism for cAMP into the matrix.

It is noteworthy that ATP-binding cassette (ABC) transporters have been found to work synergistically with PDEs regulating both local and global cAMP level [137, 138]. Recently one of the ABC B subfamily members has been shown to export cAMP in *D. discoideum* [139]. Several evolutionarily related ABC B subfamily proteins have been found localized on the IMM [140, 141]. It would be interesting to test whether any of these ABC transporters might contribute to the matrix cAMP transport.

sACs can be activated by direct Ca^2+^ binding in a dose-dependent manner [13], demonstrating a potential crosstalk between these two most common second messengers [32, 142, 143]. In cells that lack the active transporting mechanism for cAMP into the matrix, Ca^2+^ may relay the cytosolic cAMP signaling to the matrix and activate sAC wherein [96]. Thus the intra-mitochondrial Ca^2+^ and cAMP signaling may act synergistically in regulating energy metabolism [15, 32, 96, 144]. Whether Ca^2+^, or other messengers, affords coordination between cytosolic and intra-mitochondrial cAMP signaling, awaits further investigation. And to what extent a crosstalk between cAMP and other signaling is achieved to regulate mitochondrial behaviors remains to be explored.

### Potential applications to the treatment of mitochondrial dysfunction diseases

Given the essential role of mitochondrial cAMP signaling in mitochondrial dynamics, biogenesis and metabolism, it is not surprising that its misregulation can cause various disorders, particularly in cells and tissues with high-energy demand. For instance, displacement of AKAP121 increases ROS production, induces apoptosis and triggers cardiac hypertrophy in transgenic rodents [145]. Loss of AKAP and decline of mitochondrial PKA signaling are believed to contribute to the etiology of several brain degenerative disease models [146]. In *Drosophila,* the misregulation of the intra-mitochondrial cAMP signaling impairs mitochondrial biogenesis and triggers neurodegeneration [103]. On the other hand, manipulating mitochondrial cAMP signaling might provide a handle to modulate energy metabolism and control cell death, thereby offering potential avenues for managing mitochondrial diseases and neuromuscular diseases associated with mitochondrial dysfunctions. Several compounds that modulate cAMP-PKA signaling globally, including PDE inhibitors and PKA inhibitors (H89), have shown promise in treating inflammation, diabetes and cardiovascular disorders [35, 147, 148]. However, their application to modulating mitochondrial cAMP signaling locally has to be cautioned. In particular, specific delivery of drugs into mitochondrial compartments is necessary to avoid broad activation or inhibition of cellular cAMP signaling. A lipophilic cation, triphenylphosphonium (TPP^+^), which preferentially accumulates in the mitochondrial matrix can effectively target antioxidants and metabolic-modulating compounds to mitochondria [149]. Peptides containing a unique aromatic-cationic sequence motif also concentrate on the mitochondria inner membrane [149, 150]. It would be interesting to test whether these molecules can be used to deliver cAMP signaling-modulating compounds to specific mitochondrial compartments, and thereby to improve mitochondrial functions without interfering with other cAMP signaling processes.

## Abbreviations

cAMP: Cyclic adenosine 3′,5′-monophosphate
ATP: Adenosine triphosphate
OXPHOS: Oxidative phosphorylation
mtDNA: Mitochondrial DNA
ETC: Electron transport chain
OMM: Outer mitochondrial membrane
AC: Adenylyl cyclase
TmAC: Transmembrane adenylyl cyclase
sAC: Soluble adenylyl cyclase
PKA: Protein kinase A
CREB: cAMP response element binding protein
PLC: Phospholipase C
PKC: Protein kinase C
PI3K: Phosphoinositide 3-kinase
IP3: Inositol trisphosphate
PDE: Cyclic nucleotide phosphodiesterase
AKAP: A-kinase anchoring protein
IMS: Intermembrane space
IMM: Inner mitochondrial membrane
TOM: Translocase of the outer membrane
AAC: ADP/ATP carrier
MDV: Mitochondrion-derived vesicles
Drp1: Dynamin-related protein 1
Mfn2: Mitofusin 2
OPA1: Optic atrophy 1
MAPK: Mitogen-activated protein kinases
GSK-3β: Glycogen synthase kinase 3β
SKIP: Sphingosine kinase interacting protein
FRET: Fluorescence resonance energy transfer
TCA: Tricarboxylic acid
BiFC: Bimolecular fluorescence complementation
3-MST: 3-Mercaptopyruvate sulfurtransferase
CBS: cystathionine β-synthase
CSE: Cystathionine g-lyase
H_2_S: Hydrogen sulfide
COXIV-1: Cytochrome *c* oxidase subunit IV isoform 1
COX: Cytochrome *c* oxidase
IF1: ATPase inhibitory factor 1
ROS: Reactive oxygen species
PGC-1α: Peroxisome proliferator-activated receptor gamma co-activator 1α
NRF: Nuclear respiratory factor
mtTFA: Mitochondrial transcription factor A
ABC: ATP-binding cassette
TPP^+^: Triphenylphosphonium cation
